# Supplementary material for: Recent European marine heatwaves are unprecedented but not unexpected
Source: Commun Earth Environ. 2025 Oct 7;6(1):792. doi: 10.1038/s43247-025-02802-3 (PMC12504108; doi:10.1038/s43247-025-02802-3)
Supplement: Supplementary file 2 — Supplementary Information [file 43247_2025_2802_MOESM2_ESM.pdf]

Supplementary Information for "Recent European marine heatwaves are unprecedented but not unexpected"

Jamie R. C. Atkins[1,2], Adam A. Scaife[1,3], Jennifer A. Graham[4], Jonathan Tinker[3], Paul R. Halloran[1].

[1]*Faculty of Environment, Science and Economy, University of Exeter, Exeter, UK.*

[2]*Institute for Marine and Atmospheric Research, Utrecht University, Utrecht, The Netherlands.*

[3]*UK Met Office Hadley Centre, Exeter, UK.*

[4]*Centre for Environment, Fisheries and Aquaculture Science (Cefas), Lowestoft, UK.*

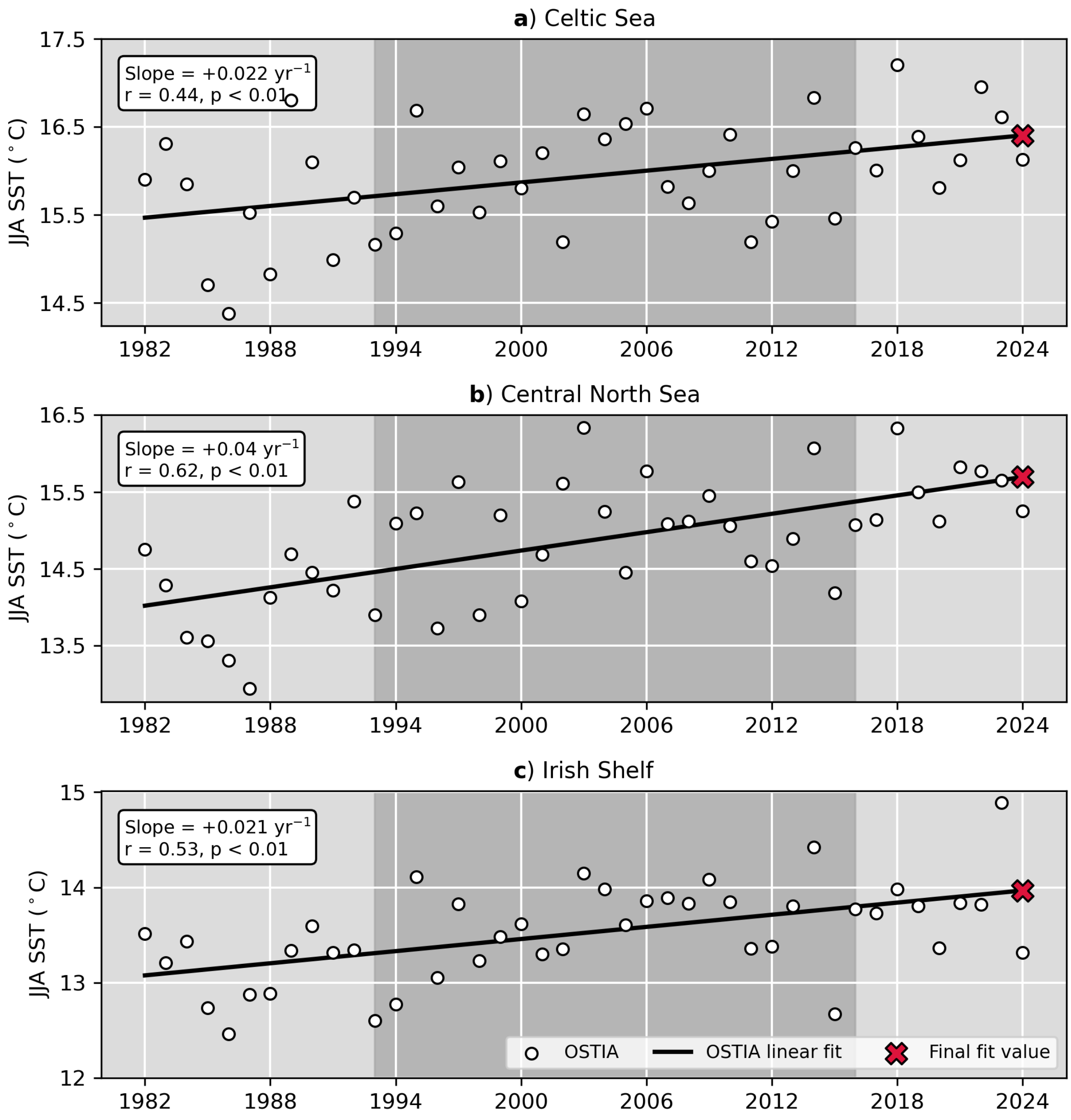

Figure S1 – European North-West shelf seas warming rates. OSTIA JJA SST (black scatter markers) across the period 1982-2024 in the Celtic Sea (a), central North Sea (b) and Irish Shelf (c) sub-regions. The OSTIA linear regression fit is shown by the black line, and the associated slope, r and *p* values for the fit are annotated by text in each panel. Dark grey shading highlights the 1993-2016 hindcast period. Red markers mark the final point on the OSTIA linear fit (used for "last differencing"; see Methods).

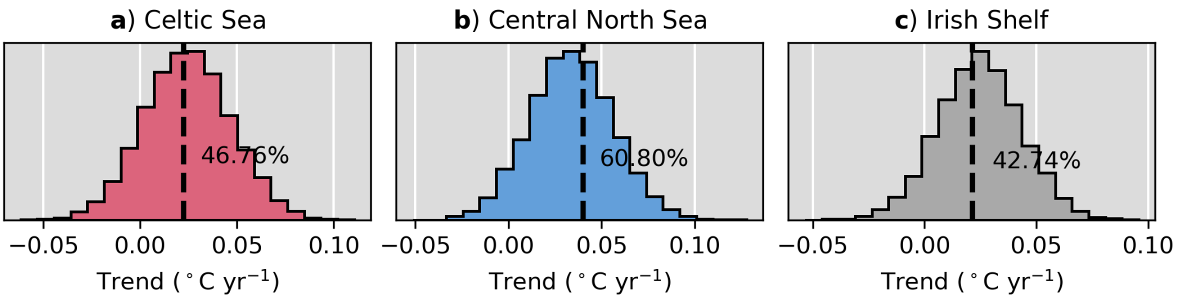

Figure S2 – Modelled vs. observed NWS SST warming trends. Frequency distributions of proxy model SST trends (n = 10,000) in the Celtic Sea (a), central North Sea (b) and Irish Shelf (c) sub-regions. The observed SST trend is marked by black dashed lines. The percentile value at which the observed statistic lies within the modelled distribution is marked by text annotation. The proxy model SST timeseries (n = 10,000), from which the trends are calculated and the modelled distributions built, are generated by sampling the full model ensemble by bootstrapping (with replacement) across the hindcast period. The timeseries span the common period between model and observations (i.e. 1993-2016 hindcast period).

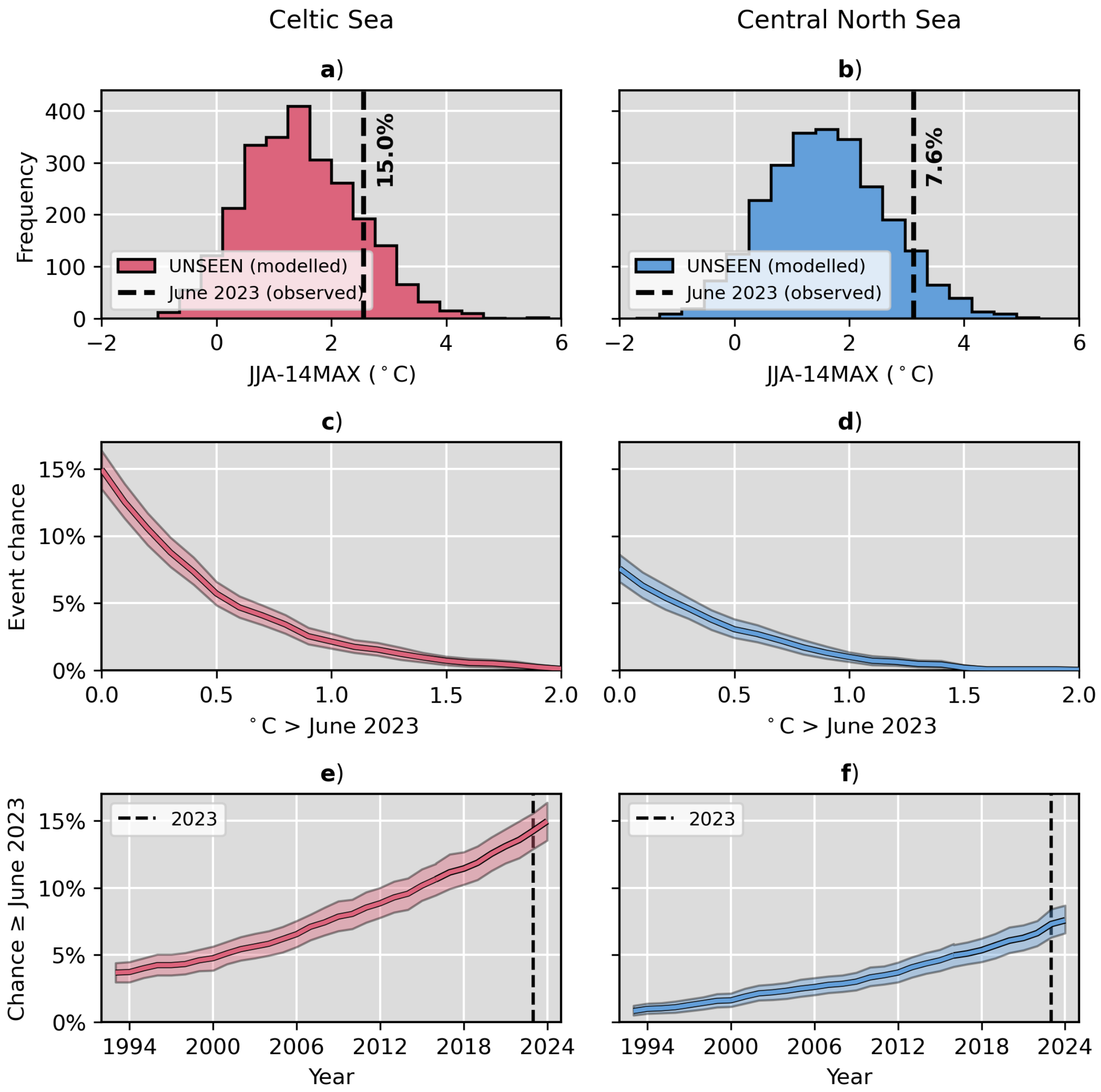

Figure S3 – UNSEEN MHWs exceeding the June 2023 event (reference-year detrended using model ensemble trend). For the Celtic Sea (red; panel column a, c, e) and central North Sea (blue; panel column b, d, f): UNSEEN distribution of modelled JJA-14MAX events (a, b), UNSEEN estimate of event chance as a function of degrees Celsius above June 2023 (c, d) and UNSEEN estimate of June 2023 event chance as a function of time since 1993 (e, f). Observed peak 14-day rolling mean SST in June 2023 is marked by the black dashed lines in a) and b), and the probability of this event in the UNSEEN distribution (i.e. 100% minus percentile rank) is shown by text annotation. In c) – f), probabilities are also calculated relative to observational estimates, shading represents the 95% confidence interval calculated by bootstrapping (n = 1000). The UNSEEN reference-year detrending approach for 2024 climate (here, calculating the trend from the model ensemble mean, extrapolated to 2024; see Methods) is applied in all panels except e) and f) where probabilities are calculated from distributions which are reference-year detrended to reflect the climate of each year in turn. The vertical black dashed lines in e) and f) mark the year 2023, i.e. when the June 2023 event actually occurred.
